# Agent-Based Modeling of Virtual Tumors Reveals the Critical Influence of Microenvironmental Complexity on Immunotherapy Efficacy

**DOI:** 10.3390/cancers16172942

**Published:** 2024-08-23

**Authors:** Yixuan Wang, Daniel R. Bergman, Erica Trujillo, Anthony A. Fernald, Lie Li, Alexander T. Pearson, Randy F. Sweis, Trachette L. Jackson

**Affiliations:** 1 Department of Mathematics, University of Michigan, Ann Arbor, MI 48109, USA; shirlynw@umich.edu (Y.W.); bergmand@umich.edu (D.R.B.); 2 Department of Medicine, Section of Hematology/Oncology, The University of Chicago, Chicago, IL 60637, USA; erica.trujillo815@gmail.com (E.T.); afernald@bsd.uchicago.edu (A.A.F.); lli2@uchicagomedicine.org (L.L.); alexander.pearson@uchicagomedicine.org (A.T.P.); rsweis@bsd.uchicago.edu (R.F.S.)

**Keywords:** agent-based model, ordinary differential equation, immune checkpoint inhibition, tumor antigenicity, bladder cancer, cytotoxic T lymphocyte, Fas/Fas ligand, perforin/granzyme

## Abstract

**Simple Summary:**

Immune checkpoint inhibitors (ICIs) are cancer immunotherapeutics that reinvigorate immune cells’ ability to attack tumor cells. Despite remarkable results in some patients, ICIs do not demonstrate the same efficacy across all individuals. In this study, we present the first side-by-side comparison of an agent-based model (ABM) with an ordinary differential equation (ODE) model for ICIs targeting the PD-1/PD-L1 immune checkpoint. We consider tumor cells of high and low antigenicity and two distinct immune-cell kill mechanisms. Using key parameters calibrated from mouse bladder cancer studies, we simulate virtual tumors using both models. Our research identifies crucial tumor-immune characteristics that influence the efficacy of ICIs. By exploring the unique spatial insights provided by the ABM, we underscore the importance of considering the spatial complexity of the tumor microenvironment in mathematical models of ICIs, potentially paving the way for more effective cancer treatments.

**Abstract:**

Since the introduction of the first immune checkpoint inhibitor (ICI), immunotherapy has changed the landscape of molecular therapeutics for cancers. However, ICIs do not work equally well on all cancers and for all patients. There has been a growing interest in using mathematical and computational models to optimize clinical responses. Ordinary differential equations (ODEs) have been widely used for mechanistic modeling in immuno-oncology and immunotherapy. They allow rapid simulations of temporal changes in the cellular and molecular populations involved. Nonetheless, ODEs cannot describe the spatial structure in the tumor microenvironment or quantify the influence of spatially-dependent characteristics of tumor-immune dynamics. For these reasons, agent-based models (ABMs) have gained popularity because they can model more detailed phenotypic and spatial heterogeneity that better reflect the complexity seen in vivo. In the context of anti-PD-1 ICIs, we compare treatment outcomes simulated from an ODE model and an ABM to show the importance of including spatial components in computational models of cancer immunotherapy. We consider tumor cells of high and low antigenicity and two distinct cytotoxic T lymphocyte (CTL) killing mechanisms. The preferred mechanism differs based on the antigenicity of tumor cells. Our ABM reveals varied phenotypic shifts within the tumor and spatial organization of tumor and CTLs despite similarities in key immune parameters, initial simulation conditions, and early temporal trajectories of the cell populations.

## 1. Introduction

The versatility of mathematical and computational models has made them an increasingly crucial tool in biomedical research. Models create abstract and simplified representations of real-world phenomena, allowing researchers to gain deeper insights into inherently complex biological processes. These biologically driven and carefully calibrated models extend beyond purely theoretical pursuits. They can shed light on important underlying mechanisms, predict emergent patterns [1], test therapeutic strategies [2], and even inform the design of clinical trials [3,4]. Over the past decade, immunotherapy has established a new paradigm for the treatment of cancer [5,6]. Immunotherapy is fundamentally different from traditional first-line therapies, such as radiation and chemotherapy [7]. By harnessing the immune system’s power, immunotherapy overcomes immunosuppression induced by a tumor and its microenvironment, allowing the immune system to target and kill cancer cells [5,6]. Among the various immunotherapy methods such as direct immune modulators, monoclonal antibodies, oncolytic viruses, adoptive cell therapy and vaccines [6,8,9], immune checkpoint inhibitors (ICIs) have garnered significant attention. ICIs are a class of immunotherapeutics that reinvigorate the killing activities of immune cells by blocking the activation of inhibitory immunoreceptors [7,10]. They have shown remarkable results for many patients. However, ICI monotherapy’s low overall response rates and difficulty enhancing patients’ responses with combination therapy in many cancers present an ongoing challenge to clinicians [8,11,12].

ICIs aim to revitalize cytotoxic T lymphocytes (CTLs), which are a key component of the adaptive immune system and major killers of pathogens and neoplastic cells [13,14,15,16]. Adding further complexity to the varied antitumor immune responses is the fact that cytotoxic T lymphocytes (CTLs) execute their cell-killing function via at least two distinct mechanisms [17,18]. The first process is mediated by perforin and granzymes. Perforin facilitates the formation of pores in the target cell membrane, which allows granzymes to access the target cell cytoplasm to induce apoptosis [17,19,20]. The second process is through the Fas pathway. FasL, a type II transmembrane protein upregulated on CTLs, can engage Fas on the target cell to trigger apoptosis of the target cell [17,21]. Evidence showed that the perforin/granzyme-mediated process happens faster than the FasL-mediated process [17]. In an in vitro study, perforin-mediated killing was completed within thirty minutes, whereas FasL-based killing was detected no sooner than two hours after the tumor cell was conjugated with CTL [21]. Evidence also showed that the switch from fast to slow killing is related to the decreasing presence of antigens [22]. Although the connections between distinct CTL killing mechanisms are not fully understood, we find it important to consider the immune system’s varied responses towards tumor cells with different antigenicity and to integrate them into our computational models.

With the increasing amount of high-throughput data to analyze and the plethora of treatment strategies to test, reliable and cost-efficient computational modeling becomes an indispensable tool in studies of cancer immunotherapy [23,24]. To explain the wide variations of patient responses, quantify the influence of spatial complexity in the tumor microenvironment (TME), and predict which patients are most likely to respond well to ICIs, we build mathematical and computational models for the ICIs targeting the PD-1/PD-L1 immune checkpoint. Differential equation-based models and agent-based models (ABMs) are popular modeling approaches for cancer treatments. Ordinary differential equation (ODE) models describe the temporal evolution of populations of cells or molecules through a set of coupled mathematical equations. In contrast, partial differential equation (PDE) models describe spatial-temporal dynamics using densities of cells or concentration gradients. ODE-based models in immuno-oncology include those that represent general tumor-immune dynamics [25], oncolytic virus therapy [26], anti-PD-1 immune checkpoint inhibitors [27], resistance to dendritic-cell vaccines [28] and so on. PDE-based models include but are not limited to [29,30], which focus on therapies involving anti-PD1 ICIs. On the other hand, an ABM simulates how individual entities, such as cells and molecules, move and interact with each other and with the environment. Many ABMs have been developed to model the TME and cancer immune response [31]. We previously developed the first ODE model building on the works of [25,27], and subsequently the first ABM for anti-PD-1 immune checkpoint blockade therapy with consideration of tumor cells of different antigenicity and the two aforementioned CTL killing mechanisms [32,33]. However, the ABM in [33] also includes the anti-FGFR3 small molecule inhibitors. The ABM in this paper is adapted from [33] to focus on the activity of the PD-1/PD-L1 immune checkpoint and the two CTL killing mechanisms like in the ODE model.

By comparing fundamentally different modeling approaches, an ODE model, and an ABM of the same biological process, we make use of their strengths and also explore their limitations. ODEs allow rapid simulations and thorough diagnostics. On the other hand, ABMs reflect the discrete nature of biology better [24] and can reveal emergent behaviors that would be missed in a purely equation-based approach. While our previous work analyzed the ODE model in detail to identify important characteristics of the tumor-immune landscape that have the largest impact on the outcomes of immune checkpoint blockade, this paper centers on examining what aspects of the tumor-immune dynamics both the ODEs and ABM can describe and what unique insights the ABM can offer due to the integration of the spatial elements. By comparing and contrasting the ABM and the ODE model and using the immune checkpoint inhibitors as an example, we will also discuss the balance between model tractability, model complexity, and computational efficiency when building models for cancer immunotherapy. The paper is structured as follows: Section 2 explains our experimental and modeling methods; Section 3 provides a detailed description of the simulation results, which serve as the basis for the discussions in Section 4 on the preclinical and clinical implications of our models and a comparison of the two modeling strategies. Section 5 concludes the paper.

## 2. Materials and Methods

**Figure 1:**
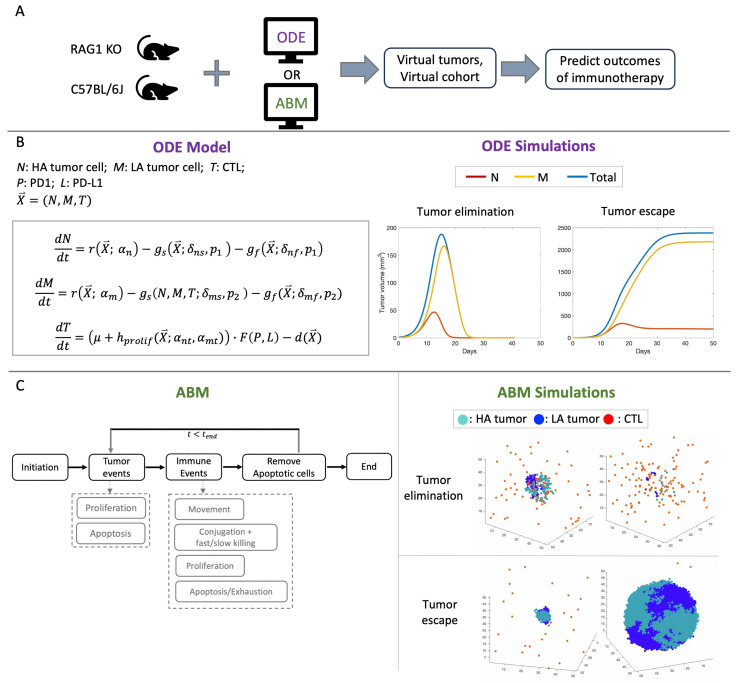
(**A**) Simulation pipeline: In vivo data of RAG1 KO and C57BL/6J mice calibrate key parameters in the ODE model and the ABM to simulate virtual tumors and virtual cohort and predict therapeutic outcomes of anti-PD1 immune checkpoint inhibitors (ICIs). (**B**) Formulation of the ODE model and the temporal trajectories of tumor volumes after immune checkpoint blockade in “elimination” and “escape” cases. HA: high antigen, LA: low antigen. *r*: logistic growth of the tumor; $g_{f}$: fast killing of the tumor cells; $g_{s}$: slow killing of the tumor cells; μ: constant recruitment of CTLs per day; $h_{prolif}$: antigen-stimulated proliferation of CTLs; *F*: immune suppression by the PD1-PD-L1 complex; *d*: death/exhaustion of CTLs. See [32] for exact formulation of the ODE model. See Table A2 for descriptions of key ODE parameters shown in this figure. (**C**) Simplified flowchart of the ABM and simulations of tumor elimination and tumor escape after immune checkpoint blockade. At each time step, each tumor cell can either proliferate or undergo apoptosis. Each CTL undergoes one of the four events: movement, conjugation with tumor cells to attach via fast or slow killing, proliferation, apoptosis, or exhaustion.

Key steps of our experimental, modeling, and analytical pipeline are outlined in Figure 1. We use in vivo data to build a biologically informed ODE model and ABM to simulate virtual tumors in a virtual cohort with diverse tumor-immune characteristics and predict individual responses to immune checkpoint blockade therapy. Further details about the experiments, model formulation, and model calibration are provided in this section.

### 2.1. Computational Models

We compare two mathematical models to describe the tumor-immune dynamics with an active or blocked PD1/PD-L1 immune checkpoint. The first formulation is an ODE model that tracks the temporal changes in the number of tumor cells, CTLs, and concentration of PD-1 and PD-L1. The details of this ODE model are previously published in [32]. The second formulation is a three-dimensional, on-lattice ABM in which tumor cells and immune cells are modeled as autonomous agents interacting with each other and the TME. Like in the ODE model, the ABM has three types of cells: high-antigen (HA) tumor cells, low-antigen (LA) tumor cells, and CTLs. Cells in the ABM occupy lattice sites. Tumor cells are immobile, while CTLs are mobile. At each time step, tumor cells can proliferate or undergo apoptosis. The proliferation of tumor cells slows down due to contact inhibition [34] because tumor cells are immobile in this ABM. Here, we only simulate the virtual tumor until it escapes or metastasizes into nearby blood vessels. Hence, simulations stop when the tumor cells exceed the maximum number allowed or too many tumor cells have reached the boundaries of the TME lattice. The model employs an immune stimulatory factor (ISF), a construct representing the combined effect of factors that each tumor cell secretes into the local neighborhood of the tumor microenvironment. The level of ISF expression depends on the cell’s antigenicity. LA tumor cells secrete a fraction of ISF compared to HA tumor cells.

In the ABM, CTLs are recruited from the lattice boundaries at a constant rate, independent of tumor size. At each time step, a CTL can execute one of the following actions: proliferation, apoptosis or exhaustion, movement, or conjugation. The proliferation rate of CTLs depends on both a base rate and the concentration of ISFs in the surrounding environment and is also affected by contact inhibition. CTL exhaustion occurs as a result of extended antigen exposure [35,36,37]. CTL apoptosis also arises naturally [37]. Since both dead and exhausted CTLs lose effector functions, the apoptosis and exhaustion of CTLs are combined into a single event in the ABM. The direction of CTL movement is influenced by the concentration gradient of ISF in the TME, i.e., CTLs are more likely to move in the direction of higher ISF. Once CTLs conjugate with a tumor cell, they attempt to destroy it via fast or slow killing. In our previous ABM [33], HA tumor cells are only killed via the fast mechanism, and the LA tumor cells are only killed via the slow mechanism. We relax this restriction, adding the probability of fast killing for both HA and LA tumor cells. This allows maximum modeling flexibility and allows us to assess the importance of considering the two killing mechanisms in tumor-immune dynamics. The assumption in the baseline parameter set is that CTLs kill HA tumor cells preferentially via the fast mechanism and kill LA tumor cells preferentially via the slow mechanism. In both the ABM and the ODE model, an active PD-1/PD-L1 immune checkpoint inhibits the recruitment and antigen-mediated proliferation rates of CTLs in the TME. In both models, we categorize therapeutic outcomes into “elimination”, “dormancy”, and “escape”, which correspond to the three phases of immunoediting: elimination, equilibrium, and escape [38,39].

### 2.2. Description of Experiments

For mouse experiments, 6–8 week old female RAG1 KO and C57BL/6J mice were obtained from The Jackson Laboratory. Mice were housed in a specific pathogen-free animal facility at the University of Chicago and used in accordance with the animal experimental guidelines set by the University of Chicago Animal Care and Use Committee (IACUC). All experimental animal procedures were approved by the IACUC.

The MB49 cell line is a chemical carcinogen-induced urothelial carcinoma cell line derived from a male C57BL/lcrf-a’ mouse. Cells were maintained at 37 °C with 5% CO_2_ in DMEM supplemented with 10% heat-inactivated FCS, penicillin, and streptomycin. $1\times 10^{6}$ MB49 tumor cells were subcutaneously injected into the flank of RAG1 KO (n = 27) or C57Bl/6J (n = 24). Four types of MB49 cells with different expression levels of the model antigen SIY (SIYRYYGL) were used: Zs green (no SIY), L14 (low SIY), H1 (high SIY), and a mix of L14 and H1 cells with 1:1 ratio. Each type of MB49 cell were injected into five to seven mice of each strain. Mice that died or had tumors with more than 50% ulceration were excluded from the data used for model calibration. On Days 7, 10, 12, 14, 17, and 19, tumors were measured three-dimensionally using a digital caliper. Tumor volume was calculated using L × W × H. All mice were sacrificed on Day 20 in accordance with IACUC guidelines for humane endpoints. Tumors were harvested and digested in 10% FBS/RPMI. Single cell suspensions were filtered through a 100 μM cell strainer and stained with antibodies to PD-1, CD69, CD3, CD19, LAG3, Ki67, CD4, CD44, CD45, CD8a, SIY, CD62L, Foxp3, and Live/Dead Viability Dye Zombie NIR. CTLs were analyzed using flow cytometry. The number of CD8 cells is directly measured and the CTL density within the tumor, i.e., the number of CD8 cells per ${\mathrm{mm}}^{3}$ of tumor is calculated.

### 2.3. Estimation of Model Parameters and Construction of Virtual Tumors

**Figure 2:**
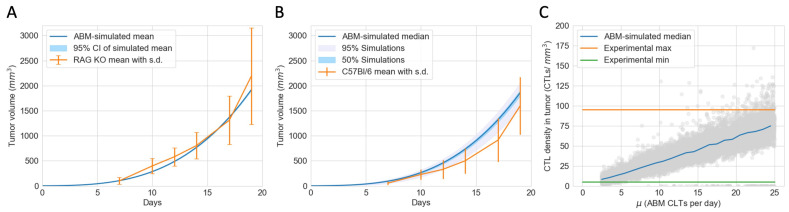
The ABM is calibrated to closely reflect experimental data of mice with an active PD-1/PD-L1 immune checkpoint. (**A**) Tumor volumes of immunocompromised RAG1 KO mice (in orange) and virtual tumors in the absence of an immune system (in blue) from Day 0 to Day 19. Error bar: standard deviation of RAG1 KO tumor volumes. Very narrow shaded region: 95% confidence interval of the simulated mean. (**B**) Tumor volumes of immunocompetent C57Bl/6J mice (in orange) and a virtual cohort comprising 12,000 simulated tumors with immune responses (in blue) from Day 0 to Day 19. Error bar: standard deviation of CB57Bl/6J tumor volumes. Shaded grey region: 5 to 100 percentile of simulated tumor volumes on each day, chosen to include most tumors that escape pre-treatment. Shaded blue region: 25 to 75 percentile of simulated tumor volumes on each day. (**C**) Grey circles: simulated endpoint CTL densities in virtual tumors at different CTL recruitment rates (μ). Blue line: median CTL density of virtual tumors with μ values in each integer bin. Orange and green lines: maximum and minimum CTL densities in experimental data of CB57Bl/6J mice on Day 19.

**Figure A1:**
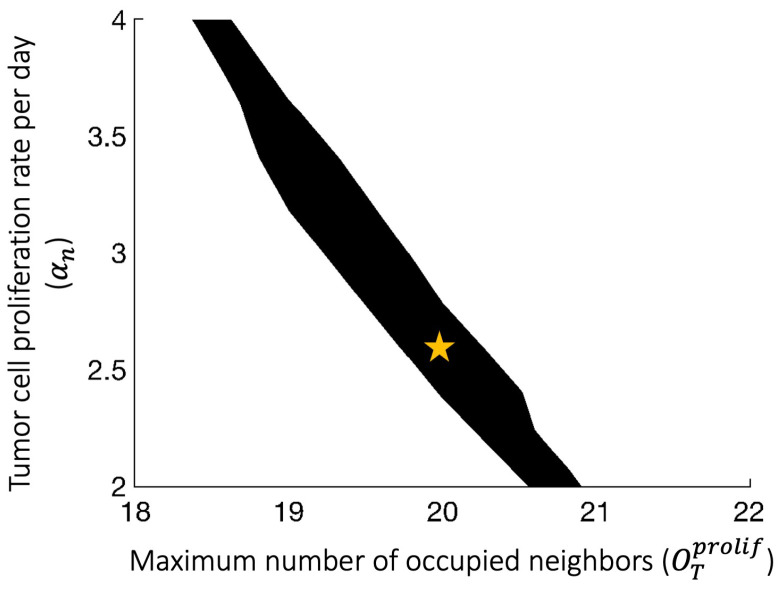
Using the Surrogate Modeling for Reconstructing Parameter Surfaces (SMoRe ParS) method [41] to construct the admissible parameter region for which $\alpha_{n}$ and $O_{T}^{prolif}$ accurately capture the RAG KO tumor growth data. Yellow star: baseline parameters used to generate the cohort of virtual tumors.

To convert tumor volume to number of tumor cells, we assume that 1 ${\mathrm{mm}}^{3}$ of tumor is equivalent to $10^{6}$ tumor cells [40]. Given this conversion rate and the initial conditions of the experiments and the ABM simulations, each ABM tumor cell represents 50,000 actual tumor cells. The proliferation rate ($\alpha_{n}$) and the carrying capacity (K) of the ODEs are calibrated using a simplified ODE model without an immune system and the tumor volumes of RAG1 KO mice. The parameters were chosen to minimize the mean squared error between actual and predicted tumor volumes. The proliferation rate ($\alpha_{n}$) and the contact inhibition parameter ($O_{T}^{prolif}$) of tumor cells in the ABM were calibrated using the tumor volumes of RAG1 KO mice, and the Surrogate Modeling for Reconstructing Parameter Surfaces (SMoRe ParS) method developed by [41]. SMoRe ParS employs an ODE surrogate model to estimate ABM parameter values from experimental data. The admissible parameter region for which $\alpha_{n}$ and $O_{T}^{prolif}$ accurately capture the tumor growth data is shown in Figure A1. From this region, we select baseline values for $\alpha_{n}$ and $O_{T}^{prolif}$ so that we can focus on exploring the effects of other parameters related to the immune system and tumor antigenicity. In Figure 2A, the blue line shows the mean volume of 25 virtual tumors with calibrated $\alpha_{n}$ and $O_{T}^{prolif}$. The range of simulated tumor volumes at each time point shows little variation, and the simulated trajectory closely matches the mean tumor volume of RAG1 KO mice, as shown in orange.

Fixing the calibrated $\alpha_{n}$ and $O_{T}^{prolif}$, we then varied ten other tumor-immune characteristics using Latin Hypercube Sampling in the range given in Table 1 to construct a virtual cohort comprising 12,000 simulated TMEs. The initial conditions of the simulations are shown in Table 2. Due to the stochastic nature of the ABM and computational limitations, we cannot vary all ABM parameters. We chose ten parameters that we believed would have the most impact on therapeutic outcomes based on the most sensitive parameters in the ODE model, which describes the same biological process, and our understanding of the spatial components of the ABM. In Figure 2B, the blue line shows the median, interquartile range, and 95% simulated interval of tumor volumes up to Day 19. The orange lines show the mean and standard deviation of tumor volume of C57BL/6J mice on days when measurements were taken. The simulated trajectories lie reasonably close to the experimental data.

Based on the calculated density of CTLs within the tumor in C57BL/6J mice at the endpoint of Day 19, we estimated each ABM CTL represents 2175 actual CTL cells. This scale was calculated, and the range of the CTL recruitment rate in the ABM was chosen so that the range of simulated CTL densities on Day 19 in the virtual cohort matches the range observed experimentally, as illustrated in Figure 2C. The green and orange lines show the observed minimum and maximum CTL density in C57BL/6J mice on Day 19. The grey dots show the endpoint CTL density of each virtual mouse, and the blue line shows the median CTL density for each integer interval of the CTL recruitment rate (e.g., 2–3, 19–20, etc.). The calibrated parameters are shown in Table 3. All other ABM parameters are in Table A1.

## 3. Results

### 3.1. Immunotherapy Efficacy Widely Varies in Virtual Cohort with Indistinguishable Pretreatment Tumor Growth Patterns

**Figure 3:**
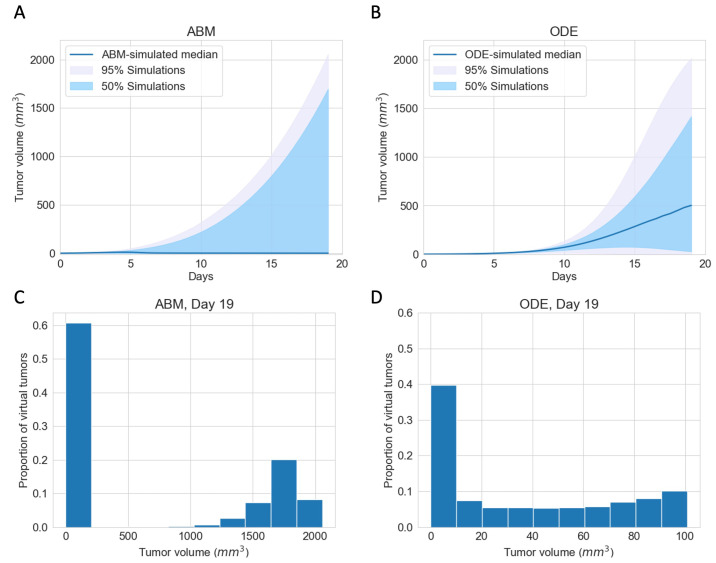
ABM and ODE virtual cohort simulations show a wide range of efficacies for immune checkpoint blockade therapy. (**A**) ABM simulations of virtual cohort response to ICI. (**B**) ODE simulations of virtual cohort response to ICI. Blue line: simulated median. Shaded grey region: 5 to 100 percentile of simulated tumor volumes on each day. Shaded blue region: 25 to 75 percentile of simulated tumor volumes on each day. (**C**) Histogram of ABM-simulated tumor volumes on Day 19. (**D**) Histogram of ODE-simulated tumor volumes on Day 19.

To explore the best-case scenarios of checkpoint blockade therapy in the same virtual cohort as in Figure 2B for which pre-treatment growth patterns are similar, we simulated completely blocking the PD-1 PD-L1 immune checkpoint in both the ODE model and the ABM. Figure 3A,B show the median, 95%, and 50% simulated interval of tumor volume, with (A) corresponding to the ABM simulations and (B) corresponding to the ODE simulations. Both ODE and ABM are able to capture a wide range of treatment outcomes after immune checkpoint blockade, as shown by similar 95% simulated interval (shaded grey) and 50% simulated interval (shaded light blue). Tumor status after treatment ranges from elimination to escape by Day 19. This result contrasts with the tight 95% simulated interval of tumor growths in Figure 2B, for the same virtual cohort of mice with an active immune checkpoint. This implies that tumors that grow similarly pre-treatment can have drastically different therapeutic outcomes after immune checkpoint blockade therapy.

In (A) and (B), we notice stark differences in the median trajectories of the tumor volume. In the ODE model, the median tumor volume achieves a moderate size, which we characterize as dormancy, by Day 19. In contrast, in the ABM, the median tumor volume is small enough to be described as eliminated by Day 19. A closer look at tumor volumes on Day 19 reveals that most tumors resolve into an elimination or escape steady state outcome much faster in the ABM than in the ODE. Figure 3C,D show the distribution of tumor volume on Day 19. Tumors in ODE simulations range from 0 to 2000 ${\mathrm{mm}}^{3}$, whereas tumors in the ABM simulations are either close to 0 ${\mathrm{mm}}^{3}$ or above $1000\;{\mathrm{mm}}^{3}$, with no in-between cases. The lack of intermediate tumor sizes on Day 19 in the ABM suggests that a typical virtual tumor either gets eliminated or escapes by Day 19.

### 3.2. Initial Phenotypic Composition Dictates Composition and Volume of Tumor after Checkpoint Blockade Therapy

In virtual clones with identical tumor and immune characteristics, ODE and ABM simulations show that different initial percentages of LA tumor cells result in different outcomes of checkpoint blockade therapy. Our models’ baseline assumption is that LA tumor cells have a survival advantage over HA tumor cells. In the ODE model and the ABM, HA tumor cells are more likely to be killed via the fast mechanism, and LA tumor cells are more likely to be killed via the slow mechanism. Moreover, in the ABM, CTLs are more likely to move towards HA tumor cells than LA tumor cells, increasing the likelihood of CTL conjugation with HA tumor cells and HA cell clearance.

**Figure 4:**
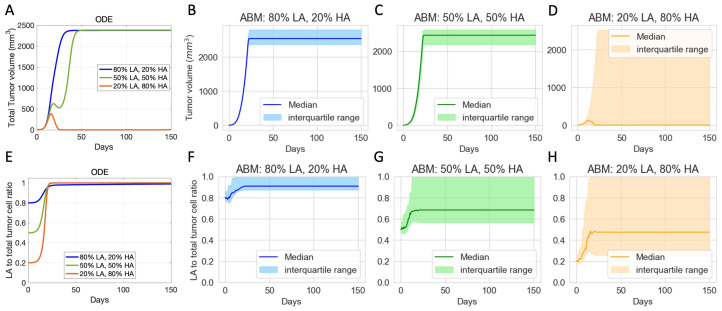
Virtual clones with identical tumor-immune characteristics but different initial tumor compositions show varied long-term responses to checkpoint blockade therapy. (**A**) Volumes of ODE-simulated virtual tumors. (**B**–**D**) Volumes of ABM-simulated virtual tumors. (**E**) Ratio of low-antigen (LA) tumor cells to total tumor cells in ODE-simulated virtual tumors. (**F**–**H**) Ratio of LA tumor cell to total tumor cell in ABM-simulated virtual tumors. Colors show different compositions of the initial tumor. Blue: 80% LA tumor cells, 20% HA tumor cell. Green: 50% LA, 50% LA. Orange: 20% LA, 80% HA. ODE parameters for virtual clones in (**A**,**E**): $\alpha_{nt}=0.32,\;\mu =2\times 10^{4}$. ABM parameters for virtual clones in (**B**–**D**) and (**F**–**H**): $\alpha_{nt}=0.32,\;\mu =15$. Other parameters are set at baseline.

For the chosen set of parameters, Figure 4A shows that, using the ODE model, if the initial tumor is 50% or 80% LA tumor cells, the tumor grows to the maximum possible volume. Using the ABM model, Figure 4B,C show that the tumor escapes, but there are slight variations in the long-term steady-state tumor volume. This variation in final tumor volume in panels B and C is likely due to one of our ABM’s stopping criteria. The ABM stops and marks the tumor as “escape” once sufficient ABM tumor cells reach the boundary of the TME, and this might happen at different times for each of these tumors that escaped. Therefore, the tumors in panels B and C have no qualitative differences in terms of tumor volume. With 20% LA tumor cells initially in the ODE model, the tumor gets eliminated by Day 28, as shown by the orange line in Figure 4A. In the ABM, an initial LA ratio of 20% makes tumor elimination possible but not guaranteed. In this case, the outcome ranges from tumor elimination to tumor escape across simulations, with a wide range of possible steady-state tumor sizes in between, as shown by the wide interquartile range in Figure 4D.

In terms of tumor composition, Figure 4E–H show that across all ODE and ABM simulations with different initial tumor compositions, the final tumor is always more LA-dominant than the initial tumor, even in the cases where the tumor shrinks. This result reflects the survival advantages that LA tumor cells have in our models. It also suggests that checkpoint blockade therapy can reduce the tumor size but increase the proportion of LA tumor cells in the resulting tumor, which can impact the results of other subsequent immunotherapies. While both ODE and ABM simulations show a trend toward increased LA ratio, there are notable differences in the final tumor compositions. In the ODE simulations, the final tumor is consistently 100% LA tumor cells as shown in Figure 4E. However, in the ABM, the final tumor can exhibit a wider range of LA ratios, with the range expanding as the initial ratio of LA tumor cells decreases (Figure 4F–H).

### 3.3. Therapeutic Outcomes Are Correlated with Key Immune Parameters

**Figure 5:**
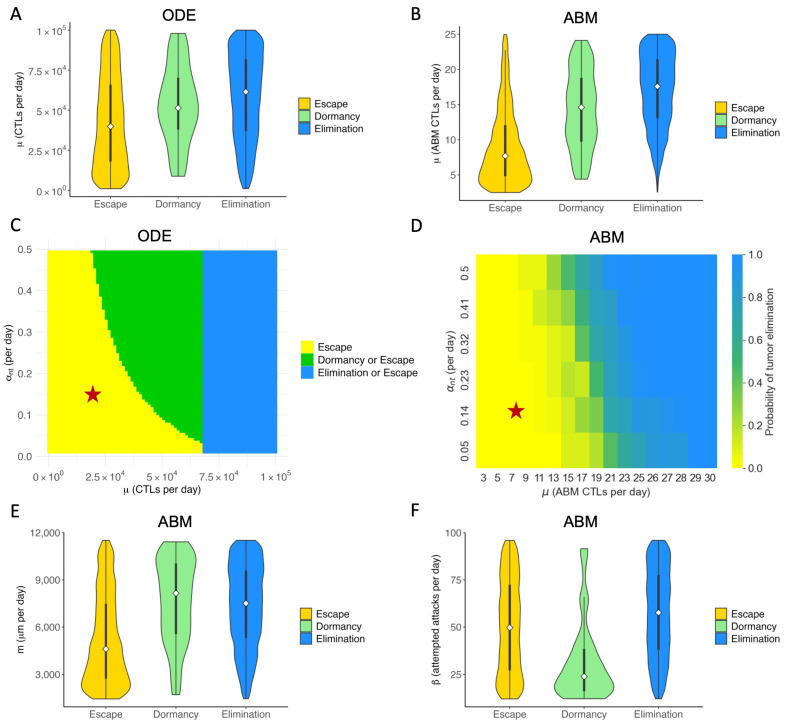
Key immune parameters in ABM and ODE models are correlated with outcomes of immune checkpoint blockade. Colors in all sub-panels represent long-term (t≥150 days) outcomes of checkpoint blockade therapy. Elimination (blue): tumor size $<0.1\;{\mathrm{mm}}^{3}$; Dormancy (green): $0.1<\mathrm{tumor}\;\mathrm{size}<500\;{\mathrm{mm}}^{3}$; Escape (yellow): tumor size > $500\;{\mathrm{mm}}^{3}$. (**A**) ODE: Violin plot of the distribution of CTL recruitment rate (μ) in the virtual cohort associated with each outcome, with the shape showing probability density, the white circle showing the median, and the black lines showing the interquartile range. (**B**) ABM: Violin plot of the distribution of μ in the virtual cohort associated with each outcome. (**C**) ODE: Two-parameter bifurcation diagram of μ and max antigen-stimulated CTL proliferation rate ($\alpha_{mt}$) on steady-state tumor size. Red star: baseline parameters. (**D**) ABM: Probability of tumor elimination at each μ-$\alpha_{nt}$ combination. Colormap shows the probability of tumor elimination ranging from 0 (yellow) to 1 (blue). (**E**) ABM: CTL movement rate (*m*). (**F**) ABM: CTL conjugation rate (β).

We explore what tumor or immune characteristics are most correlated with therapeutic outcomes of checkpoint blockade in the ODE model and the ABM model. In particular, we are interested in knowing whether there are similar conclusions for parameters common to both models and how spatial parameters unique to the ABM relate to the outcomes. In our extensive analysis of the ODE model [32], we determined that the CTL recruitment rate (μ) is the most important immune parameter for achieving tumor reduction and elimination. The ABM indeed corroborates this result. Figure 5A shows the distribution, median, and interquartile range of the CTL recruitment rate in the ODE model in the virtual cohort associated with each therapeutic outcome. Figure 5B shows a similar graph for μ in the ABM. The median and interquartile range of μ associated with tumors that eventually get eliminated are higher than those of escape cases. The ABM shows a more prominent separation of the distribution of μ between elimination and escape cases, as the 75 percentile in the escape cases is lower than the 25 percentile in the elimination cases. This suggests the CTL recruitment rate might be even more predictive of treatment outcomes in the ABM than in the ODE model.

In our analysis of the ODE model, we examined the combined effect of varying both CTL recruitment rate and maximum antigen-mediated CTL proliferation rate. The parameter with an equivalent effect in the ABM is the maximum ISF-stimulated proliferation rate of CTLs ($\alpha_{nt}$). Figure 5C shows the two-parameter bifurcation diagram of μ and $\alpha_{nt}$ in the ODE model. An analogous approach was used in the ABM to capture the effect of the ODE bifurcation analysis. Figure 5D depicts the probability of tumor elimination in the virtual cohort of mice as μ and $\alpha_{nt}$ vary. Blue represents a 100% chance of elimination, and yellow represents a 0% chance of tumor elimination. Due to the stochastic nature of the ABM, different runs of the same set of parameters yield different tumor outcomes, resulting in the gradient of colors between blue and yellow in panel D. Green regions in Figure 5D thus represent a non-zero probability of tumor elimination. Both C and D show that our baseline case lies in the region of parameter space where the tumor escapes to carrying capacity with certainty. For virtual TMEs with a low μ value, only increasing $\alpha_{nt}$ is ineffective for reducing tumor volume in the long term. The most efficient way to reduce tumor volume at equilibrium is by increasing $\alpha_{nt}$ and μ simultaneously, landing in the green region where the tumor can be eliminated. Increasing μ sufficiently can eliminate the tumor with certainty.

**Figure A2:**
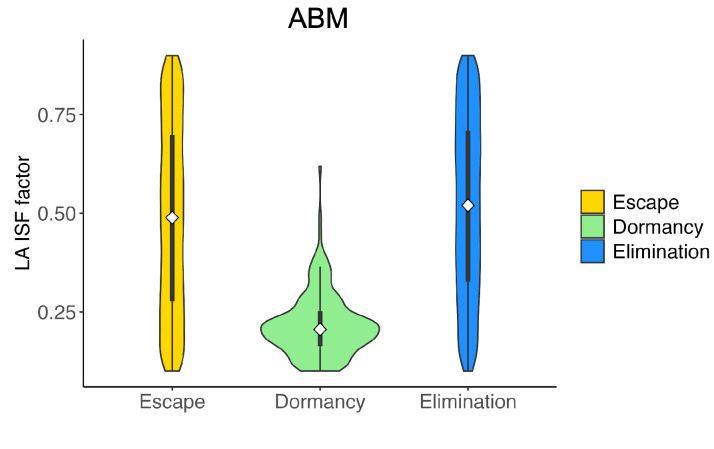
Violin plot of the distribution of LA ISF factor ($r_{\mathrm{ISF}}$) in the virtual cohort associated with each outcome, with the shape showing probability density, the white circle showing the median, and the black lines showing the interquartile range. Elimination (blue): tumor size $<0.1\;{\mathrm{mm}}^{3}$; Dormancy (green): $0.1<\mathrm{tumor}\;\mathrm{size}<500\;{\mathrm{mm}}^{3}$; Escape (yellow): tumor size $>500\;{\mathrm{mm}}^{3}$.

Figure 5E,F show the distribution of spatial parameters of tumors associated with each outcome. Figure 5E shows that escape cases generally have a much lower T cell movement rate (*m*) than elimination and dormancy cases. A low T cell movement rate, as depicted in Figure 5E, has significant consequences. It hampers the ability of CTLs to reach tumor cells fast enough to prevent the tumor from spreading to the lattice’s edge and escaping. Moreover, in the ABM cohort, only a small number of tumors have not been eliminated or escaped by Day 150, therefore being marked as dormant. Figure 5F shows that most dormancy cases have a low conjugation rate (β) between tumor and CTLs. The low conjugation rate is a significant factor in tumor dormancy. It leads to reduced interaction, prolonging the time to reach a stable term equilibrium, as shown in Figure 5F. We observe a similar pattern with the LA-ISF factor ($r_{\mathrm{ISF}}$), the ratio of ISF secreted by LA tumor cells compared to HA tumor cells, as seen in Figure A2. As CTLs are more likely to move towards regions with high ISF, a low $r_{\mathrm{ISF}}$ makes it harder for CTLs to locate and conjugate with LA tumor cells. Therefore, a low $r_{\mathrm{ISF}}$ has a similar effect as a low β: both cause the tumor to stay dormant for longer.

### 3.4. ABM Reveals Spatial and Phenotypic Heterogeneity Despite Similar Temporal Tumor and Immune Growth Patterns

**Figure 6:**
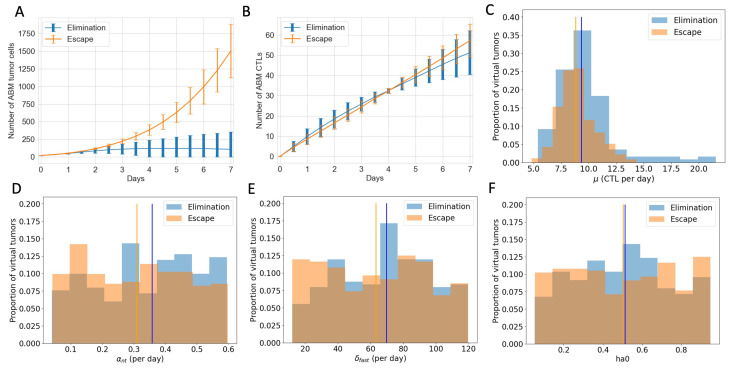
Virtual tumors with a similar number of CTLs in the TME up to Day 7 show diverging tumor control outcomes after checkpoint blockade. Blue: tumor elimination; orange: tumor escape. (**A**) Number of ABM tumor cells up to Day 7. Lines show the mean, and error bars show the standard deviation of tumor volume in each group. (**B**) Number of ABM CTLs up to Day 7. (**C**) Histogram of CTL recruitment rates (μ) in “elimination” and “escape” groups, respectively, with the y-value normalized by the total number of virtual tumors in that group. Vertical lines show the median μ of CTLs in virtual tumors in each group. (**D**) Max ISF-stimulated CTL proliferation rate ($\alpha_{nt}$) (**E**) Rate of fast killing by CTLs ($\delta_{\mathrm{fast}}$) (**F**) Initial ratio of HA tumor cells to total tumor cells ($ha_{0}$).

**Figure A3:**
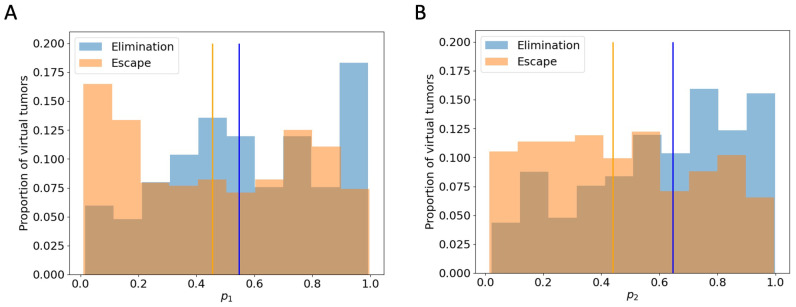
Histogram of $p_{1}$ and $p_{2}$ in“elimination” and “escape” groups respectively, with the y-value normalized by the total number of virtual tumors in that group. Vertical lines: the median μ of virtual tumors in each group. (**A**) $p_{1}$: probability of HA tumor cell death via fast killing (**B**) $p_{2}$: probability of LA tumor cell death via fast killing.

To explore what additional insights we gain using an ABM that includes spatial features, we examine tumors that are expected to grow similarly after checkpoint blockade in the ODE model but end up having different or even completely opposite therapeutic outcomes in the ABM. In particular, we analyze 603 virtual tumors with the same number of tumor cells and CTLs initially and similar CTL temporal trajectories up to Day 7. Eventually, 251 of these tumors are eliminated, and 352 escape long-term. Based on their future tumor status, we categorize these 603 tumors as “to be eliminated” and “to escape”, or “elimination” and “escape” in short. Figure 6A shows that despite the same initial condition, the number of tumor cells in the “elimination” and “escape” groups diverge quickly between Day 2 and Day 3. However, the total numbers of CTLs in the TME in both groups remain close up to Day 7, as illustrated in Figure 6B. Analysis of the ODE model in [32] identified CTL recruitment rate as the most critical parameter for predicting tumor outcomes after checkpoint blockade, followed by maximum antigen-stimulated CTL proliferation rate and fast-kill rate. Figure 6C–E show similar distributions and close medians of these key immune parameters in “elimination” and “escape” groups. In Figure 4, we saw that initial tumor compositions can greatly influence the outcomes of checkpoint blockade therapy. Nonetheless, there are no clear patterns of LA-dominance or HA-dominance in the initial tumors in either group, as illustrated by comparable distributions and close medians of the initial ratio of HA tumor cells ($ha_{0}$) in Figure 6F. With checkpoint blockade therapy, the probabilities of fast and slow killing ($p_{1},p_{2}$) were not in the top half of most sensitive parameters in terms of tumor volumes in the ODE model, although they were in the top quartile in terms of tumor composition [32]. Nonetheless, here in the ABM, $p_{1},p_{2}$ show some correlation with the therapeutic outcomes, as seen in Figure A3. Tumors with low $p_{1}$ or $p_{2}$ are more likely to escape, although there is no strong monotonic relationship between the values of $p_{1},p_{2}$ and the likelihood of tumor escape or elimination.

Given similar initial conditions, key immune parameters, and temporal trajectories of the number of CTLs, the ODE model is unable to describe or explain the disparate treatment outcomes. Therefore, we focus on parameters and features that are unique to the ABM to search for explanations.

**Figure 7:**
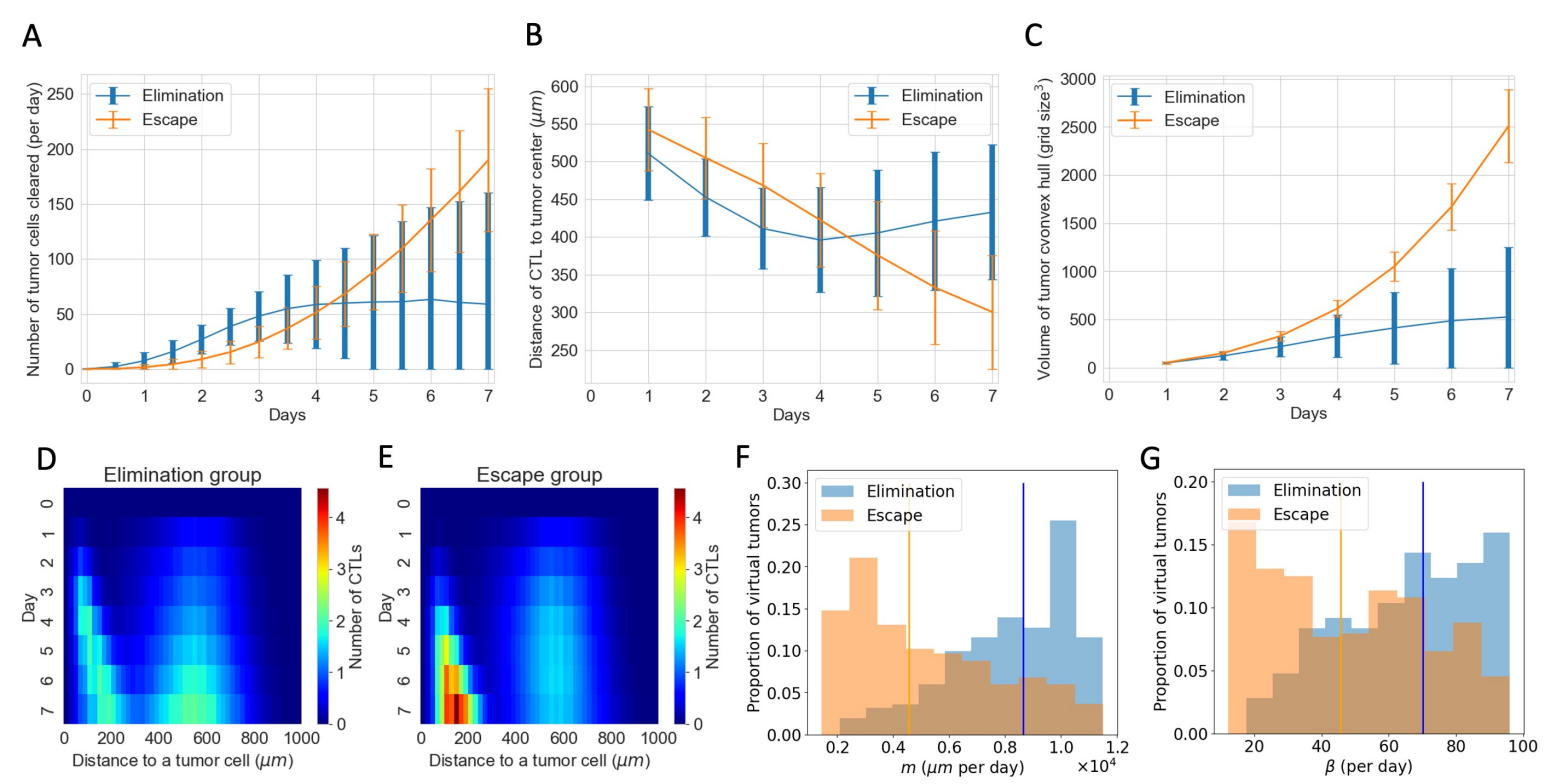
Spatial features explain diverging therapeutic outcomes after immune checkpoint blockade despite a similar total number of CTLs in the TME. (**A**) Number of tumor cells cleared by CTLs per day in “elimination” and “escape” groups. Blue: elimination; orange: escape. The line shows the mean and error bars show standard deviation in each group. (**B**) Mean distance of CTLs to the tumor center. (**C**) Volume of the tumor convex hull (**D**,**E**) *Y*-axis shows time. *X*-axis shows the distance of a CTL to a tumor cell. The color represents the mean number of CTLs at a certain distance from a tumor cell at that time point. D corresponds to the “elimination” group and E corresponds to the “escape” group. (**F**) Normalized histogram of CTL movement rate (*m*) of virtual tumors in “elimination” and “escape” groups, respectively. Vertical lines: median *m* in each group. (**G**) CTL conjugation rate (β).

Figure 6B shows the temporal evolution of the total number of CTLs in the TME. However, it does not tell us where these CTLs are and how they interact with the tumor cells. Figure 7 complements information about CTLs in the ABM. Figure 7A shows that despite the “escape” group and “elimination” group having similar numbers of CTLs, there are clearly more tumor cells cleared by CTLs per day in the “elimination” group. Figure 7B shows that on average, at each time point, CTLs in the “elimination” group are closer to the tumor center than those in the “escape” group in the first four days, allowing CTLs to be closer to tumor cells and have a higher chance of clearing tumor cells early on. Another spatial feature is the volume of the tumor convex hull, which is defined as the smallest convex shape that includes all the tumor cells. Figure 7C shows a smaller mean tumor convex hull in the “elimination” group, meaning that the tumor is more compact. The combined effect of more CTLs closer to the tumor center and a smaller convex hull is that the CTL density within the tumor is higher in the “elimination” group starting on Day 2, making conjugations and tumor cell clearance more likely. In addition to examining CTLs in relation to the tumor center, we also consider the spatial distribution of CTLs with respect to each tumor cell. Figure 7D,E show the temporal evolution of the distributions of CTLs relative to tumor cells in the ABM. The top-down direction shows the evolution of time. The *x*-axis shows the distance from a tumor cell. The color map represents the mean number of CTLs at a certain distance from a tumor cell at each time point from Day 0 to Day 7, averaged across all virtual tumors in each group. In the first four days, there are more CTLs close to tumor cells (e.g., distance <160μm) in the “elimination” group. From Day 4 onwards, although the number of CTLs near each tumor cell increases in the “escape” group, it is too late. The tumors still escape eventually.

Parameters existing in both the ABM and the ODE model show no clear distribution pattern in the “elimination” and “escape” groups in Figure 6. However, spatial parameters in the ABM that are not captured by the ODE model show distinct distribution patterns corresponding to each outcome group. This helps explain the observations about the number of tumor cells cleared per day and spatial distributions of CTLs in the “elimination” and “escape” groups in Figure 7A,B,D,E. Movement rate (*m*) and conjugation rate (β) skew to the right in the “elimination” group and to the left in the “escape” group in Figure 7F,G. Almost all virtual tumors with extremely low *m* or β values escape. On the contrary, a virtual tumor with high *m* or β values is much more likely to get eliminated than to escape. CTLs with high *m* values have higher motility and can thus co-locate with tumor cells faster. CTLs with high β values are more likely to conjugate with tumor cells at each time step. Therefore, given a similar number of CTLs, higher movement and conjugation rates of CTLs are more likely to result in favorable outcomes after checkpoint blockade therapy in the long term.

## 4. Discussion

We simulated tumor progression and the response to immune checkpoint blockade therapy in a virtual cohort using a three-dimensional, on-lattice ABM calibrated using in vivo data from bladder cancer studies in mice. Here, we present a comparison of an ODE model and an ABM for the same cancer immunotherapy: ICI for the PD-1/PD-L1 immune checkpoint. Our models reveal which tumor and immune characteristics affect the outcomes of checkpoint blockade therapy the most. While our previous work [32] analyzed the ODE models thoroughly, this paper focuses on the capabilities of the ABM. In this way, we explore what biological insights both models can provide and what additional insights the ABM offers about the spatial complexity of the TME and its impact on therapeutic outcomes. Despite the enhanced modeling capabilities, the use of ABMs also presents challenges. Therefore, we will also discuss the pros and cons of the ODE model and the ABM for modeling tumor-immune dynamics.

The ODE model and ABM predict a wide range of therapeutic responses to immune checkpoint blockade therapy in a virtual cohort with similar tumor growth pre-treatment. Both models also identify crucial immune parameters linked to the range of outcomes. Our analysis of both models underscores the pivotal role of CTL recruitment rate (μ) and maximum rate of antigen-mediated CTL proliferation ($\alpha_{nt}$) in tumor reduction or elimination. Since adoptive T-cell therapy can increase μ and therapeutic cytokines like interleukin-2 (IL-2) can increase $\alpha_{nt}$, our results in Figure 5A–D have implications on the effectiveness of combination therapy strategies. Our simulations suggest that combination therapy of anti-PD1 and adoptive T-cell transfer is effective in drastically reducing tumor size or eliminating tumors if the CTL recruitment rate can be enhanced to sufficiently high levels. Various combination therapy strategies involving anti-PD1 and tumor-infiltrating lymphocytes (TIL) or chimeric antigen receptor (CAR) T cells have shown synergistic effects in both preclinical studies and clinical trials [44,45]. Lifileucel, a TIL therapy, was recently approved for patients who received prior treatment with anti–PD1/PD-L1 antibodies [45]. Our simulations suggest another possible way to achieve a drastic reduction of tumor volume or even tumor elimination with a smaller amount of drug: combining ICIs with both adoptive T-cell transfer and cytokine-directed therapy. In this way, a patient’s parameters can move from a baseline outcome of ICI monotherapy, where the tumor escapes with certainty, to a region in parameter space where tumor elimination is possible. In fact, IL-2 treatments are often administered with other forms of immunotherapy, such as Lifileucel. Furthermore, IL-2 therapy in combination with anti-PD1/PD-L1 was shown to be feasible and tolerable, although the clinical trials to show the effectiveness of this therapy are still underway [46,47].

Both models also show the importance of considering tumor antigenicity and multiple immune-cell kill mechanisms preferentially associated with HA or LA tumor cells. Our baseline assumption was that CTLs preferentially kill HA tumor cells via the fast mechanism and LA tumor cells via the slow mechanism. Effectively, we assumed that LA tumor cells are the harder-to-treat phenotype regarding antigenicity. Using virtual clones with different initial LA to total tumor cell ratios, we showed that the less LA-dominant the initial tumor is, the better the outcomes after immune checkpoint blockade. Moreover, the final tumor was always more LA-dominant than the initial tumor. These are both consequences of CTLs killing HA tumor cells faster than LA tumor cells. Higher numbers of LA tumor cells in the resulting tumor suggest that if ICI does not eliminate the tumor, it might become a “colder” tumor, thereby affecting the responses to subsequent treatments. The shift to LA-dominance aligns with well-documented observations of immune selection for lowly antigenic tumors [48]. In the ABM, the antigenicity of the tumor cells not only determines how fast CTLs kill tumor cells once conjugation has occurred; it also greatly impacts the movement of CTLs before conjugation. CTLs gravitate towards regions with high ISF, and HA tumor cells secret higher ISF in our model. This key difference between HA and LA tumor cells underlies the impact of the CTL movement rate, conjugation rate, and LA-ISF factors on treatment outcomes.

The ABM enhances our understanding of the TME by incorporating spatial characteristics that ODEs cannot capture. This allows for more nuanced insights, revealing complexities that might be overlooked when immune parameters, initial tumor composition, and the temporal evolution of cellular populations appear similar. In both models, we observed the importance of CTL recruitment rate (μ) and max antigen-stimulated CTL proliferation rate ($\alpha_{nt}$) to tumor elimination after immune checkpoint blockade. This might seem intuitive as higher μ and $\alpha_{nt}$ results in more active CTLs in the TME, and thus, they eliminate more tumor cells. However, we chose 603 virtual tumors from ABM simulations to show that, when considering intratumoral spatial heterogeneity, tumors with similar μ and $\alpha_{nt}$ values and similar temporal trajectories of CTLs in the TME can experience drastically different fates after checkpoint blockade therapy (Figure 6 and Figure 7). In the ODE model formulation, CTLs indiscriminately target all HA or all LA. By contrast, in the ABM, immune attacks are contingent on CTLs moving toward tumor cells and successfully conjugating with them. Therefore, the movement rate of CTLs (*m*) and the conjugation rate of CTLs with tumor cells (β) prove to be crucial in determining how fast CTLs colocalize to, attack, and clear tumor cells. Virtual TMEs with high *m* and β are more likely to get tumor elimination after checkpoint blockade.

Translational data are emerging on the critical nature of spatial relationships in the immune tumor microenvironment. A multitude of factors, such as gradients of chemokines and physical features of the microenvironment, have been shown to affect T cell movement [49]. In a melanoma mouse study, adoptive T-cell therapy successfully controlled tumor growth in some cases but failed in others. The T-cell infiltration and motility were higher in responders relative to non-responders, as evidenced by increased speed and distance traveled of T cells [50]. An in vitro study of melanoma showed that varied ICI responses were not merely due to differences in tumor structure or proportion of cell types. Physical proximity and contact frequency between CTLs and tumor cells also significantly differed between responders and nonresponders of ICIs [51]. Among many ongoing efforts to develop therapeutics to enhance T-cell motility and infiltration, tebentafusp, a bispecific protein consisting of an affinity-enhanced T-cell receptor fused to an anti-CD3 effector that can redirect T cells to target glycoprotein 100–positive cells [52], was FDA-approved in 2022.

With proper formulation, both ABMs and ODE models can accurately reflect biological processes. Still, they have a few fundamental differences, which lead to their respective pros and cons from the modeling perspective. ODEs model the population-level temporal dynamics of each type of cell or drug molecule, whereas ABMs model each cell as an autonomous agent. At a given time point, all cells or molecules of a single type in the ODE undergo the same changes uniformly, whereas agents in the ABM experience different events based on their location in space or what other agents surround them. The ABM captures the phenotypical heterogeneity of the three-dimensional tumor in space and the spatial activities of CTLs in the TME. This approach allows us to obtain the spatial distribution of CTLs with respect to each tumor cell and the frequency with which each CTL attacks and clears tumor cells. These individual cell-level insights explain the diverging outcomes of immune checkpoint blockade in virtual tumors despite a similar total number of CTLs in the TME. Such details cannot be obtained in continuous differential equation-based models. Thus, the ABM is undoubtedly more flexible in modeling intratumoral differences and more closely reflects complexities seen in vivo. Moreoever, the discrete and stochastic nature of ABMs, contrasted with the continuous and deterministic nature of ODEs, might have caused the ABM to wander away from locally stable equilibrium, leading to what we observed in Figure 3C,D. Tumor growths reach equilibrium faster in the ABM than in the ODE model, leading to the lack of intermediate-sized tumors on Day 19 in the ABM. The enhanced granularity and versatility of ABMs come at the cost of longer computational time and increased difficulty in parameterizing and analyzing the model. Because the ABM updates each cell individually at each time step, simulations slow down significantly when the number of tumor cells increases exponentially. Thus, simulating tumor and immune dynamics at a realistic scale is computationally prohibitive. ABMs generally have many more parameters than the ODE model, making parametrization of the model challenging. In the ODE, we used sensitivity analysis to determine which parameters impact the tumor outcome most and focused our calibration and analytical efforts on those. Sensitivity analysis of ABM parameters, though possible [53,54], is no trivial task. Future avenues of exploration include using machine learning to overcome the shortcomings of ABMs. In our upcoming work, we plan to combine this ABM with machine learning algorithms to predict the tumor-immune landscape after immune checkpoint blockade. This can make simulating larger virtual cohorts or a larger number of cells more feasible. With future developments in efficient simulations and global sensitivity analysis of ABMs, we are also interested in exploring more regions of the parameters space and comparing their impact on the TME with what we observe in this paper.

## 5. Conclusions

We presented the first side-by-side comparison of an ABM with an ODE model for ICIs targeting the PD1/PD-L1 immune checkpoint. We simulated the responses to immune checkpoint blockade therapy in a virtual cohort with diverse tumor-immune characteristics. In particular, we emphasized the importance of including spatial components in mathematical models of immunotherapy by elucidating the additional insights that the ABM provided regarding the spatial complexity of the TME and their impact on therapeutic outcomes. Our computational method can efficiently enhance the discovery of key spatial elements, inform biomarker development, and validate findings from ongoing clinical data. Even though our model was built for ICIs and was calibrated with in vivo bladder cancer data, our modeling framework and methodology can be applied to other cancers or other forms of cancer immunotherapy.

## Figures and Tables

**Table 1:** Parameters varied in the ABM.

Name	Description	Values (Baseline)	Source
μ	CTL recruitment rate	2.5–25 (8) ABM CTL d^−1^	Calibrated
$ha_{0}$	Initial ratio of HA tumor cells to total tumor cells	0.05–0.95 (0.5) d^−1^	Estimated [33]
$\alpha_{nt}$	Max ISF stimulated CTL proliferation rate	0.04–1.00 (0.15) d^−1^	Estimated [25,27,42]
$\delta_{\mathrm{fast}}$	Fast kill rate	12–120 (48) d^−1^	Estimated [33]
$p_{1}$	Probability of fast killing for HA	0–1 (0.92)	Assumed
$p_{2}$	Probability of fast killing for LA	0–1 (0.33)	Assumed
*m*	CTL movement rate	1440–11,520 (2880) μm d^−1^	Estimated [33]
β	CTL Conjugation rate with tumor cells	12–96 (28.8) d^−1^	[33,43]
$a_{\mathrm{reach}}$	Immune stimulatory factor reach	60–200 (100) μm	Estimated [33]
$r_{\mathrm{ISF}}$	ISF expression by LA tumor cells compared to HA tumor cells	0.1–0.9 (0.5)	Assumed [33]

**Table 2:** Initial conditions.

Name	Description	Value
$N_{0}$	Total Tumor cells	20 ABM cells
$T_{0}$	CTLs	0 ABM cells
$ha_{0}$	Initial ratio of HA tumor cells to total tumor cells	0.05–0.95

**Table 3:** ABM parameters calibrated from experimental data.

Name	Description	Values (Baseline)
$\alpha_{n}$	Proliferation rate of tumor cells	2.6 d^−1^
$O_{T}^{\mathrm{prolif}}$	Maximum number of occupied neighbors that still allows tumor cell proliferation (out of 26)	20 cells
μ	CTL recruitment rate	2.5–25 (8) ABM CTL d^−1^

## Data Availability

The analysis was performed using the code available in the repository https://github.com/shirlynwy/ABM_ICI_BladderCancer, accessed on 14 August 2024. Further inquiries can be directed to the corresponding author.

## Abbreviations

The following abbreviations are used in this manuscript:

ABM	agent-based model
CTL	cytotoxic T lymphocyte
FDA	US Food & Drug Administration
FasL	Fas ligand
FGFR3	fibroblast growth factor receptor 3
HA	high antigen
ICI	immune checkpoint inhibitor
IL-2	interleukin-2
ISF	immune stimulatory factor
LA	low antigen
ODE	ordinary differential equation
PD-1	programmed cell death protein 1
PD-L1	programmed death-ligand 1
TME	tumor microenvironment

## Appendix B

**Table A1:** All ABM parameters.

Name	Description	Value (s)	Source/Notes
Tumor Cell Parameters			
$\alpha_{n}$	proliferation rate	2.6 d^−1^	Calibrated
$O_{T}^{\mathrm{prolif}}$	Maximum number of occupied neighbors that still allows tumor cell proliferation (out of 26)	20	Calibrated
$\delta_{n}$	Apoptosis rate	0.05 d^−1^	Estimated [33]
$K_{\max}$	Maximum total number of tumor cells allowed	60,000	Assumed
$K_{\mathrm{edge}}$	Maximum number of tumor cells allowed to touch the boundary	36	Assumed
Immune Cell Parameters			
μ	Tumor-induced recruitment to TME	8 d^−1^	Calibrated
$\alpha_{t}$	Base proliferation rate	0 d^−1^	Assumed
$\alpha_{nt}$	Max ISF-stimulated CTL proliferation rate	0.15 d^−1^	[25,27,42]
$\delta_{t}$	Apoptosis rate	0.05 d^−1^	[25,27]
β	Immune cell conjugation rate	28.8 d^−1^	[33,43]
*m*	Movement rate	2880 μm d^−1^	Estimated [33]
$n_{\mathrm{move}}$	Number of consecutive movement steps attempted when an immune cell moves	4	Estimated [33]
$\delta_{\mathrm{exhaust}}$	CTL exhaustion rate	0.01 d^−1^	Estimated
Immune Stimulatory Factor Parameters			
$r_{\mathrm{ISF}}$	ISF expression by LA tumor cells compared to HA tumor cells	0.5	Assumed [33]
$\gamma_{I}$	EC50 for ISF stimulation of CTL proliferation	10	Assumed
$n_{I}$	Hill coefficient for ISF stimulation of CTL proliferation	2	Estimated [33]
$f_{I}$	Factor determining maximal possible increase to immune proliferation due to ISF	2.5	Estimated [33]
$\gamma_{m}$	EC50 for magnitude of ISF gradient affecting immune cell movement along gradient	2	Estimated [33]
$n_{m}$	Hill coefficient for magnitude of ISF gradient affecting immune cell movement along gradient	2	Estimated [33]
$a_{\mathrm{reach}}$	Maximum reach of ISF from one tumor cell in any one direction	5	Estimated [33]
Cell-kill Parameters			
$\delta_{\mathrm{slow}}$	Slow-killing rate	12 d^−1^	[21]
$\delta_{\mathrm{fast}}$	Fast-killing rate	48 d^−1^	[21]
$p_{1}$	Probability of HA tumor cell death via fast killing	0.92	Assumed [32]
$p_{2}$	Probability of HA tumor cell death via fast killing	0.33	Assumed [32]
PD-1/PD-L1 Parameters			
$\rho_{P}$	Concentration of PD-1 on CTLs	0.6426 n M	[55]
$k_{f_{5}}$	Association rate of PD-1-PD-L1 reaction	100 nM^−1^ d^−1^	[56]
$k_{r_{5}}$	Dissociation rate of PD-1-PD-L1 reaction	$8.25\times 10^{5}$ d^−1^	[56]
$\gamma_{e}$	EC50 of PD-1-PD-L1 complex effects on immune cells	$7.53\times 10^{-4}$ n M	Computed [33]
Miscellaneous Parameters			
Δt	Tumor update duration	15 min	Chosen
$\Delta t_{\mathrm{imm}}$	Immune update duration	7.5 min	Chosen
$L_{G0}$	Length of time in $G_{0}$ during which cells cannot proliferate	9 h	[57]
*h*	Distance between adjacent voxels	20 μm	One cell width [33]
$O_{I}^{\mathrm{prolif}}$	Maximum number of occupied neighbors that still allows immune cell proliferation (out of 26)	22	Assumption [33]
$O_{\max}^{\mathrm{move}}$	Maximum number of occupied neighbors that still allows movement (out of 26)	25	Assumption [33]

**Table A2:** Selective ODE parameters.

Name	Description	Value (Baseline)	Units	Source
$\alpha_{n}$	Proliferation rate of high antigen tumor cells	0.498	per day	Calibrated
$\alpha_{m}$	Proliferation rate of low antigen tumor cells	0.498	per day	Calibrated
K	Carrying capacity for tumor cells	$2.394\times 10^{9}$	# of cells	Calibrated
$\delta_{ns}$	Maximum CTL-induced death rate of high antigen tumor cells via the slow killing mechanism	1–12 (4)	per day	Estimated ([58])
$\delta_{ms}$	Maximum CTL-induced death rate of low antigen tumor cells via the slow killing mechanism	1–12 (4)	per day	Estimated ([58])
$\delta_{nf}$	CTL-induced death rate of high antigen tumor cells via the fast-killing mechanism	$10^{-8}$ to $10^{-6}$ $\left(2.5\times 10^{-7}\right)$	per cell per day	Estimated ([42])
$\delta_{mf}$	CTL-induced death rate of low antigen tumor cells via the fast-killing mechanism	$10^{-8}$ to $10^{-6}$ $\left(2.5\times 10^{-7}\right)$	per cell per day	Estimated ([42])
μ	Activation and recruitment rate of T cells	$5\times 10^{3}$ to $1.5\times 10^{5}$ ($2\times 10^{4}$)	# per day	Estimated
$p_{1}$	Probability of high antigen tumor cells death via the fast-killing mechanism	0 to 1 (0.92)	dimensionless	Estimated
$p_{2}$	Probability of low antigen tumor cells death via the fast-killing mechanism	0 to 1 (0.33)	dimensionless	Estimated
$\alpha_{nt}$	Maximum rate of CTL proliferation activated by N cells	0 to 0.5 (0.15)	per day	Estimated
$\alpha_{mt}$	Maximum rate of CTL proliferation activated by M cells	0 to 0.5 (0.15)	per day	Estimated
